# Nanoprocessing of layered crystalline materials by atomic force microscopy

**DOI:** 10.1186/s11671-015-0811-9

**Published:** 2015-03-12

**Authors:** Shojiro Miyake, Mei Wang

**Affiliations:** Department of Innovative System Engineering, Nippon Institute of Technology, Saitama, Japan; Department of Research and Development, OSG Corporation, Aichi, Japan

**Keywords:** Layered crystalline materials, Nanoprocessing, Microscopy, Tribology, AFM

## Abstract

By taking advantage of the mechanical anisotropy of crystalline materials, processing at a single-layer level can be realized for layered crystalline materials with periodically weak bonds. Mica (muscovite), graphite, molybdenum disulfide (MoS_2_), and boron nitride have layered structures, and there is little interaction between the cleavage planes existing in the basal planes of these materials. Moreover, it is easy to image the atoms on the basal plane, where the processed shape can be observed on the atomic level. This study reviews research evaluating the nanometer-scale wear and friction as well as the nanometer-scale mechanical processing of muscovite using atomic force microscopy (AFM). It also summarizes recent AFM results obtained by our research group regarding the atomic-scale mechanical processing of layered materials including mica, graphite, MoS_2_, and highly oriented pyrolytic graphite.

## Background

Discovering the limits of fine mechanical processing is an important subject of investigation. For example, the limit of machining is high-precision nanoscale machining, which is considered to be possible by using atomically sharp tools. The possibility of such machining has been studied using molecular dynamics.

Scanning probe microscopy (SPM) is a promising method for the nanofabrication of functional nanometer-scale engineered materials and devices [1]. For example, the study of nanofabrication by scanning tunneling microscopy (STM) was started with the nanofabrication of atomic characters, and it was later applied to various nanoscale processes [1,2]. The achievements of atomic-scale processing in machining applications are much fewer. Marking processing - a processing method for physical removal - has been tested for storage devices [3]. Several attempts have also been made to use STM for the local deposition and modification of surfaces [1,4]. In thermomechanical processing, microgrooves have been formed by indentation, and nanometer-scale grooves have been produced and used for high-density memory applications [1,5,6]. Using the direct mechanical method, atoms in Si have been mechanically displaced without disturbing the surrounding surface, creating shallow indentations with typical diameters of 3 to 10 nm [7].

Atomic force microscopy (AFM) was developed by Binnig et al. as a powerful tool to investigate the atomic-scale surface topographies of samples [8,9]. In nanotribology, the wear properties of various materials have been investigated by AFM. For instance, wear tests have been performed on ion-implanted silicon wafers, diamond-like carbon [10], ion-implanted diamond films [11], and nitrogen-containing diamond-like carbon (DLC) films [12,13]. In these tests, a high-precision square groove with an area of 1 × 1 μm^2^ and a depth of several nanometers, a line, and a space were fabricated.

Positional nanometer-scale resolution is necessary in the deposition mechanism of the tool used for forming patterns in semiconductor integrated circuits; SPM high-resolution position sensing can be applied for this purpose [14]. This can be performed by utilizing layered crystalline materials such as mica due to their anisotropic characters. The use of these layered crystalline materials with periodically weak bonds allows processing in the depth direction at a single-layer level.

On an atomic scale, mechanical processing technology is expected to be achieved in the future with AFM. To date, we have realized that the anisotropy of layered crystalline materials such as muscovite, in which the atomic structures are periodically weak, allows the following: the realization of the high-precision processing of crystalline materials using their atomic periods, layered unit processing in the depth direction, and the exploration of processing possibilities. Layered crystalline materials such as graphite, molybdenum disulfide, mica, and hexagonal boron nitride have basal planes with low levels of interaction; therefore, the surfaces of these materials are easily cleaved and observed on an atomic scale. The atomic-scale profile and friction force distribution of mica were reported by Erlandsson, J. Hu and X.D. Xiao et al. [15-18]. They found that the friction between a tungsten tip and the muscovite surface led to periodicity in the hexagonal layer of SiO_4_ units forming the cleavage plane on mica [19,20]. Otherwise, the observation of atomic images is possible at these basal planes, and a three-dimensional, high-precision criteria scale can be configured by determining the crystal lattice spacing. If a line and space pattern is formed in a mica crystal layer up to a depth of 1 nm [21], the crystal lattice spacing and a two-stepped standard subnanometer and submicrometer scale can be determined.

As a result, processing at the single-layer level can be realized by utilizing the mechanical anisotropy of layered crystalline materials with periodically weak bonds. Using AFM, we calculated the frictional force distributions and surface shapes of layered materials at the atomic level and investigated the atomic-scale wear phenomena of these materials based on the frictional force waveforms and shapes of the processed surfaces. The study revealed that wear is likely to occur in the cleavage plane spacing units [20,22]. These results have been applied to microprocessing [23].

The outline of this study is as follows: the ‘Methods’ section discusses the AFM characterization of atomic-scale wear and nanometer-scale mechanical processing of muscovite; the ‘Results and discussion’ section concentrates on the mechanical nanoprocessing of layered crystalline structures by AFM and the bending process of graphene; and the ‘Conclusions’ section concludes with an outlook.

## Methods

### Atomic-scale wear evaluation and mechanical processing methods of muscovite

**Figure 1:**
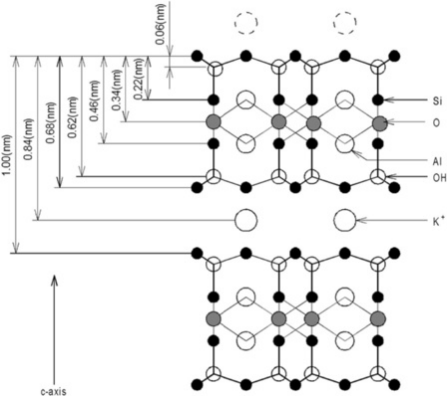
**Crystalline structure of muscovite mica.**

Mica is laminated with various elements such as Si, O, Al, OH, and K; their distances from the cleavage plane are shown in Figure 1 [19-22]. The cleavage plane is defined by a potassium layer sandwiched between two hexagonal sheets of SiO_4_ tetrahedra.

Microwear tests were performed by SPM. A silicon nitride tip with a certain fixed load was brought into contact with the muscovite surface, and a number of sliding cycles were performed. The wear groove was evaluated from the topographic change in the area subjected to sliding friction by the SPM measurement. The atomic-scale topography and atomic-scale friction force microscopy (FFM) images of mica were simultaneously observed. The atomic wear phenomenon was then evaluated based on the friction force and surface atomic topographic changes caused by sliding.

The atomic-scale micromechanical processing of muscovite - a layered crystalline material - was performed using AFM [23]. The spring’s deformation was measured by laser transformation to determine the atomic force between the tip and the surface, and reciprocal scans were performed by a piezoelectric element actuator [24]. The processed groove was evaluated on the basis of the topographic changes in the area subjected to processing.

High-precision nanoscale machining is considered to be very difficult because of energy concentration during the process. However, it is possible to reduce destruction if the tip radius of the chip is reduced, leading to stress concentration.

**Figure 2:**
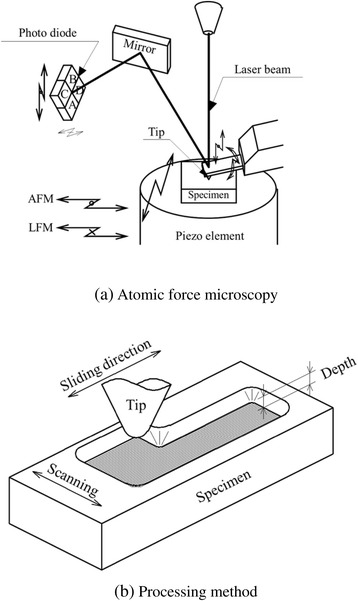
**Processing method of AFM (a) and microwear tests performed by SPM (b).**

Figure 2a shows the AFM nanoprocessing method [23], which was performed using an Si chip with a radius of less than 50 nm. The spring constant of the cantilever was calibrated as 45 N/m. From the deformation of the cantilever, the atomic force was measured using two photosensors located on the top and bottom of the cantilever. Furthermore, the process was performed by measuring the horizontal force from the torsional angle of the cantilever using the photosensors located on the right and left. To obtain the AFM image of the processed surface, the image was filtered in the frequency domain using two-dimensional fast Fourier transform (FFT), allowing the passage of a characteristic frequency.

The samples were processed in a clean booth in ambient air. When processing was performed under a humidity of over 50%, the amount of water adsorbed on the surface increased and degraded the processing. To avoid degradation due to bumps created by water absorption, processing was performed under 35% to 45% humidity. As shown in Figure 2b, microwear tests were performed by SPM. A silicon nitride tip with a certain fixed load was attached to the surface, and a number of sliding cycles were performed. The wear groove was evaluated with SPM based on the topographic change in the area subjected to sliding friction.

### Nanoindentation evaluation method for layered crystalline materials

**Figure 3:**
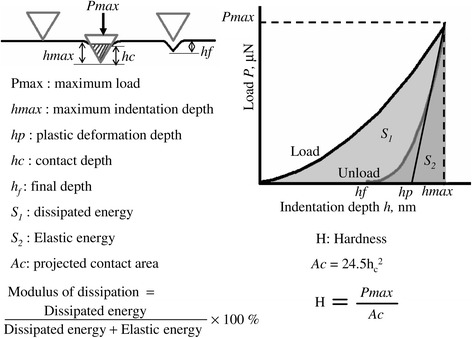
**Method for evaluating the nanoindentation hardness.**

The nanoindentation properties of layered crystalline materials were evaluated by AFM to understand their physical characteristics at a near-atomic scale. To determine the hardnesses of the layered crystalline materials, nanoindentation tests were performed by indenting a diamond-type probe (*r* = 50 to 60 nm, cube corner: 90°) [25-27] into the specimen surfaces. The load applied during the experiments varied from 10 to 340 μN, and the loading and unloading times were 5 s [28]. The resulting load penetration depth gives insight into the response of the material to mechanical stress, from which parameters such as hardness can be determined. Figure 3 shows the evaluation method. The hardness was evaluated from the plastic deformation depth, which was determined from the point of intersection of the straight line fitted to the appropriate unloading curve and the x-axis, and is assumed to be equal to the contact depth.

### Layered crystalline materials and processing methods

The nanomachining characteristics of the layered crystalline materials MoS_2_, highly oriented pyrolytic graphite (HOPG), and mica have been investigated by AFM using a superhard film tip. MoS_2_ has a hexagonal structure [28], and van der Waals forces exist between adjacent S layers. The interval between the cleavage planes is 0.616 nm. HOPG has a structure similar to that of a single crystal, and the interval between the graphite layers is 0.34 nm. The mica structure has stacks of O, Al, OH, and K, with a K layer sandwiched between the SiO_4_ cleavage planes. The layer period is 0.7 nm for the SiO_4_ layer and 1 nm for the K layer, and van der Waals forces exist between the K and SiO_4_ layers.

A DLC-coated Si tip with a tip radius of less than 50 nm was used as a tool for the nanoprocessing of the layered crystalline materials. Upon loading a certain atomic force on the DLC-coated Si tip, nanoprocessing was performed by controlling the tip-scanning path with a computer. The following section gives an example of MoS_2_ processing to obtain latticed grooves. The depth of the grooves was about 0.6 nm, which approximately corresponds to the MoS_2_ cleavage plane interval of 0.616 nm; thus, single-layer processing can be realized. Compared with MoS_2_, mica can be comparatively easily processed at the layer-unit scale. In contrast, HOPG is difficult to process using the DLC nanofilm tip because the bond strength of the basal plane is large.

**Figure 4:**
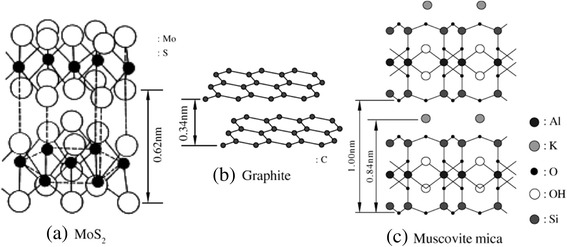
**Crystalline structures of layered materials: MoS**_2_ **(a) graphite (b), and muscovite (c).**

The MoS_2_ specimen has a hexagonal crystal structure, as shown in Figure 4. The layered interval between the S layer and the Mo layer is 0.154 nm. The interval between the S layers in crystalline MoS_2_ is 0.308 nm. Because the two adjoining S layers are bonded by van der Waals forces, its cleavage plane interval is 0.616 nm. At high temperatures (1,900°C to 2,500°C), HOPG is pyrolyzed into methane and ethane, and the structure of the accumulated matter is similar to that of monographite crystal. The crystalline structure of graphite is shown in Figure 4b; its layered interval with van der Waals forces was 0.34 nm.

Various elements contained in mica, including oxygen, aluminum, hydrogen, and potassium, overlap each other to form a crystalline structure, as shown in Figure 4c. SiO_4_ can be observed on the top of the layer. The K layer is sandwiched between the SiO_4_ layers in its cleavage planes. The SiO_4_ layer and the K layer adhere to each other through van der Waals forces. These layered crystalline materials are easily cleaved at their basal planes along the parallel direction because the van der Waals forces joining the layers are weak.

To investigate the deformation characteristics of various layered crystalline materials, indentation experiments were performed. A diamond tip was indented into the sample material with an auxiliary transducer installed on the AFM, and the indentation depth was simultaneously measured on the basis of the nanoscale deformation properties.

First, the dependence of processing properties on the number of reciprocating cycles and load was evaluated using diamond-coated tips. Second, to precisely process specimens, DLC-coated silicon tips with radii less than 50 nm were used. The nanometer-scale processing of lines and lattice grooves was performed by controlling the scanning line of the tip with a nanometer-scale lithographic program.

## Results and discussion

### AFM characterization of the atomic-scale wear of muscovite

The surface profile and friction force distribution were simultaneously measured by SPM. To acquire an atomic-scale image of the sample and to form a fairly restrictive low-pass filter to remove high-frequency noise, the images were filtered in the frequency domain with two-dimensional (2D) FFT (2D filtering). The measurements presented here were performed under 35% to 45% humidity in ambient air.

**Figure 5:**
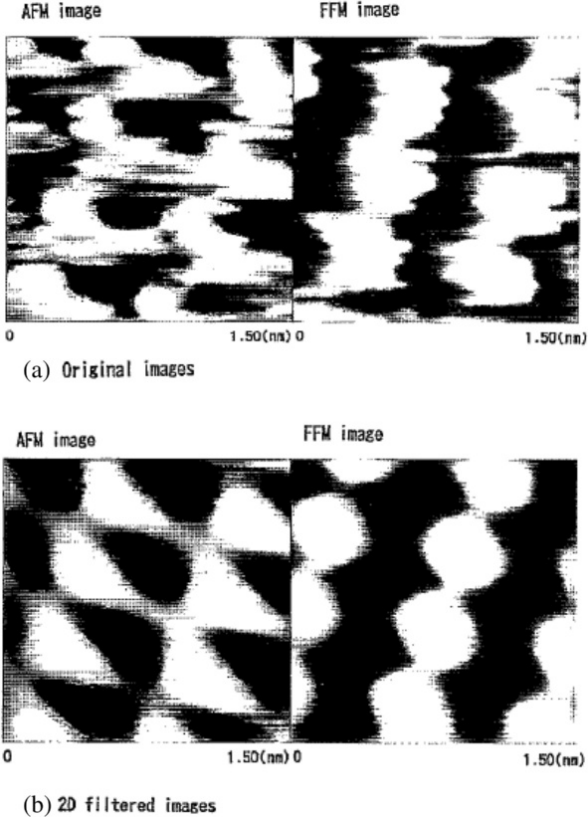
**Original images (a) and filtered images (b) of AFM and FFM images of muscovite.**

Surface images of the cleavage basal plane of muscovite observed by SPM are shown in Figure 5; (a) shows the original AFM and FFM images, and (b) shows their 2D-filtered counterparts. The surface of the cleavage plane was flat except for occasional bumps; its roughness was within 0.2 nm. The friction force and atomic force distributions had the same periodicity as the hexagons formed by SiO_4_ tetrahedra. The measured periodicity between areas of high friction force was nearly 0.5 nm, which was in agreement with the expected interval periodicity of 0.52 nm.

**Figure 6:**
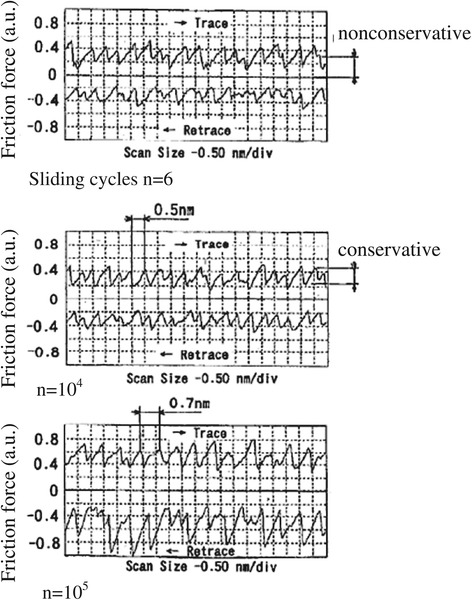
**Friction force dependence on the number of sliding cycles for muscovite.**

The dependence of the friction force of mica on the number of sliding cycles is shown in Figure 6. The friction coefficient has two components: one is conservative and periodic, while the other is nonconservative [28,29]. The change in the conservative friction force corresponds to the atomic image shown in Figure 5, whereas the nonconservative friction force corresponds to the energy that is scattered and lost. The wave profiles of conservative and nonconservative friction forces were similar for 6 and 10^4^ sliding cycles. A change in the friction force was not observed until 10^4^ sliding cycles; below this value, wear could not be observed by AFM. These results show that there is a certain range of loads that do not cause atomic-scale wear; these loads are referred to as the atomic-scale zero wear range. Wear does not occur on an atomic scale if the load and number of sliding cycles are limited to a certain range. However, at 10^5^ sliding cycles, frictional force increased 1.7 times, and the peak pitches of the conservative friction force changed from 0.5 to 0.7 nm. The change in peak pitches corresponding to the atomic image may be due to a change in the sliding direction or the sliding of the lower atomic layer after wear.

**Figure 7:**
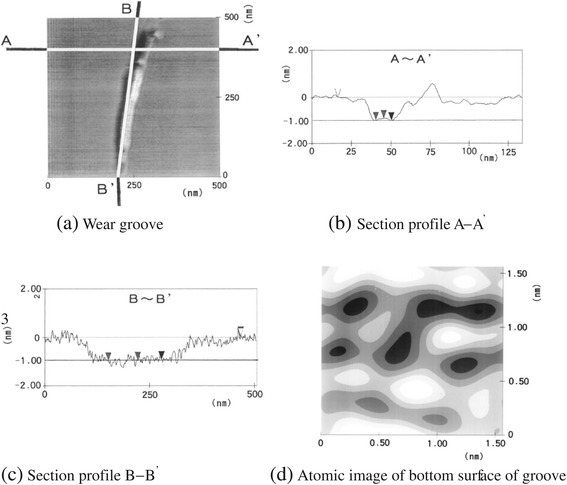
**The wear groove of mica after 10** ^5^ **sliding cycles. (a)** Wear groove, **(b)** section profile A - Aʹ, **(c)** section profile B - Bʹ, and **(d)** atomic image of the bottom surface of the wear groove.

After 10^5^ sliding cycles, a wear groove (Figure 7a) was observed. The mean depth of the wear groove was determined from the section profiles shown in Figure 7b,c to be 1.0 nm. As seen in the crystal structure of muscovite shown in Figure 1, this value corresponds to the thickness of one periodic layer, which includes two SiO_4_ layers sandwiching O, Al, and OH layers, and the K cleavage plane. The surface topography of the bottom of the wear groove is also shown in Figure 7d. The periodicity of the pitch interval corresponding to the atomic image of the basal plane was observed to be approximately 0.5 nm. These results show that a new SiO_4_ plane located one periodic layer below the top layer appeared due to wear. The bottom was not atomistically smooth and was composed of several layers of atoms. These results indicate that worn atoms adhere to the bottom surface.

### Mechanical nanoprocessing of muscovite

**Figure 8:**
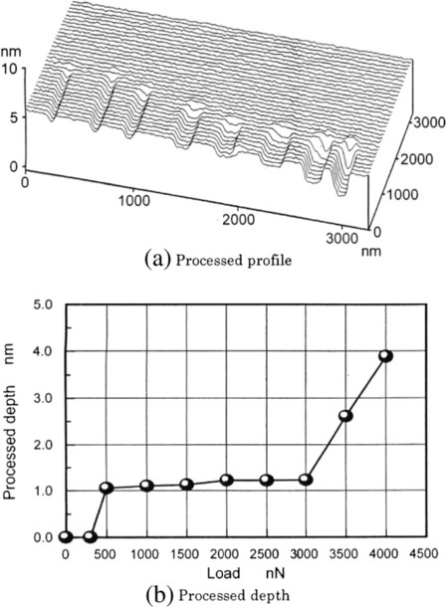
**Dependence of processing depth on load for muscovite.** Processed profile **(a)** and processed depth **(b)**.

Under a load of 100 nN, no atomic-scale-processed grooves were observed after 10^4^ sliding cycles by AFM evaluation. At loads exceeding 130 nN, grooves were formed on the damage-free mica surface. Once damage occurred on the surface, processing progressed easily. Figure 8 shows the dependence of the profile and depth of processed grooves on load after two sliding cycles. The depths of the processed grooves changed discretely with load. Processing was started with a load of 500 nN. The depths of the processed grooves were 1 nm for loads of 500 and 3,000 nN, 2.6 nm for 3,500-nN loads, and 4 nm for 4,000-nN loads. The processed depths were mainly multiples of 0.8 and 1.0 nm. The 0.8-nm depth corresponded to the distance from the top surface of SiO_4_ to the cleavage plane of potassium, while the 1.0 nm depth corresponded to the distance from the top surface of SiO_4_ to that of the next SiO_4_ beneath it. The interface between K-SiO_4_ and SiO_4_-K was weak; therefore, the removed depths of 0.8 and 1.0 nm were predominant. Potassium atoms adhered on the SiO_4_ surface were easily removed by the sliding of the tip due to the low adhesion strength between potassium and SiO_4_. Therefore, a 1-nm-deep groove with an atomically smooth bottom surface was obtained by sliding the tip several times. Larger loads damaged the layers deeper than 1 nm.

Grooves with depths of approximately 3 mm were formed under loads of 3,500 and 4,000 nN. These results indicated that 1-nm-deep mechanical processing from the surface of one cleavage plane to that of the next periodic cleavage plane was achieved by sliding the tip several times at a slightly larger load than the critical load at which the processing was started.

**Figure 9:**
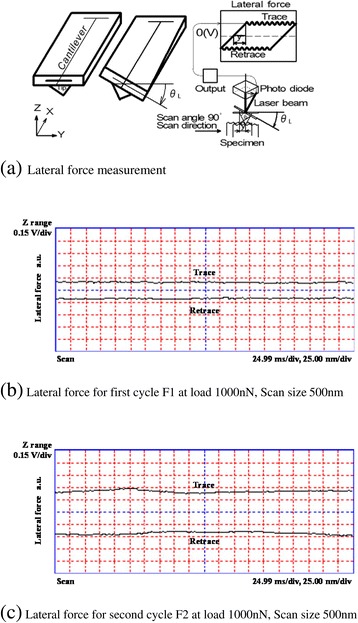
**Example of lateral force measurement.** Lateral force measurement **(a)** and lateral force for the first **(b)** and second **(c)** cycles at load 1,000 nN and scan size of 500 nm.

As shown in Figure 9a, the torsion of the tip was determined by measuring the waveform of lateral force, while processing was performed by sliding the chip in a reciprocal manner. An example measurement of the lateral force waveform is shown in Figure 9b,c, where *F*_1_ is the lateral force (resistance) of the first cycle, and *F*_2_ is that of the second cycle. The lateral force waveform was obtained at a load of 1,000 nN and a scan size of 500 nN. As shown in Figure 9b,c, the machining resistance waveforms during cutting were approximately constant.

**Figure 10:**
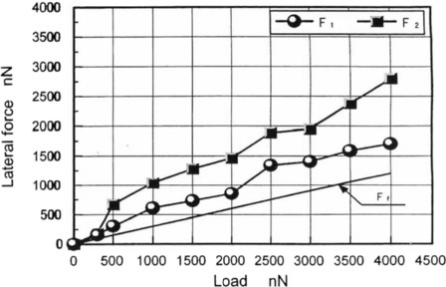
**Lateral force dependence on load.**

Figure 10 shows 1/2 of the difference in the resistance waveform obtained while sliding the chip reciprocally. Although the machining resistance tended to increase with load, its rate of increase was not constant. As shown in Figure 8b, wear depths increased at loads of 500, 3,500, and 4,000 nN. Figure 10 indicates that the sum of lateral forces *F*_1_ and *F*_2_ tended to increase with load. In other words, the increases in the horizontal loading force and machining depth almost corresponded to that in load. In the conventional wear mechanism, the resistance of single-grain machining and abrasive wear for grinding involves plowing and friction force terms [30,31]. Thus, lateral force was divided into friction force (*F*_f_), which played no role in removal, and plowing or cutting force (*F*_c_), which caused removal. The following equation is established if the coefficient of friction (μ = *F*_f_/W) is constant:

$$ F={F}_{\mathsf{f}}+{F}_{\mathsf{c}}=\upmu \mathrm{W}+{F}_{\mathsf{c}} $$

**Figure 11:**
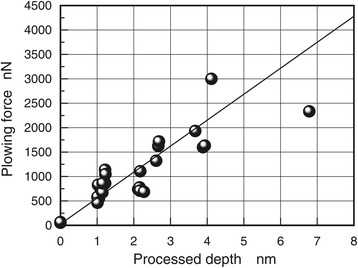
**Plowing force (** ***F***_c_ **) dependence on processed depth.**

Moreover, because *F*_c_ is zero (*F*_c_ = 0) in two-pass processing until cutting is started at a critical load of 300 nN, *F* is represented by *F* = μW. Since μ is constant at low loads, *F*_r_ is determined by drawing a line with a constant gradient (μ = a constant), as shown in Figure 10. On the other hand, *F* is larger than *F*f after cutting is started; therefore, *F*_c_ can be estimated from *F*_c_ = *F* − *F*_r_. For the lateral force of the first- and second-pass processing, the plowing force terms (*F*_c1_ and *F*_c2_) are determined first. *F*_c_ is then calculated as the sum of *F*_c1_ and *F*_c2_. Figure 11 indicates an approximately proportional relationship between plowing force and processed depth. In this case, the plowing force is about 500 nN at the processed depth of 1 nm, which corresponded to the thickness of one layer. This result shows that for atomic-scale processing, lateral force is divided into the plowing force term and the friction force term, and the former term corresponds to the processed depth.

As described in previous reports (references [30] and [31]), if the frictional force is assumed to be approximately constant, it is possible to divide the friction and cutting in an approximate fashion.

In this study, to clarify basic processing characteristics, the state of the processing (removal) at the beginning of the process was investigated. Since the processing depth fluctuates within one reciprocating processing due to the presence of residual atoms, the relationship between the processing depth and cutting resistance was determined at two reciprocating processings by measuring the frictional force at each load. At higher processing numbers, the factors related to residual atoms remaining on the surface and changes in processing groove shape became complex and represented error factors.

### Processing of multiple-stage grooves of muscovite

As seen in the dependence of mica processing on load, the surface was machined by tip sliding, forming grooves at loads at or above 130 nN. The mean depth of the processed groove was 0.7 to 1.0 nm, which corresponds to the cleavage plane spacing of muscovite. To realize the processing of 1-nm units, the processing experiment was performed with as small a load as possible.

**Figure 12:**
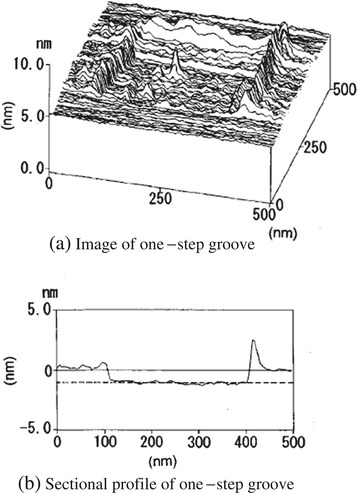
**Image (a) and sectional profile (b) of the first-step square groove processed by tip sliding.**

Figure 12a shows the processed first-step square groove with an area of 350 × 350 nm^2^ after five scans at a load of approximately 130 nN. Figure 12b shows the cross-sectional profile of the processed square groove. The mean depth of the processed groove was nearly 1.0 nm. This value corresponds to the distance between the SiO_4_ layers in muscovite. Swells of processed debris were observed at both edges of the processed groove.

**Figure 13:**
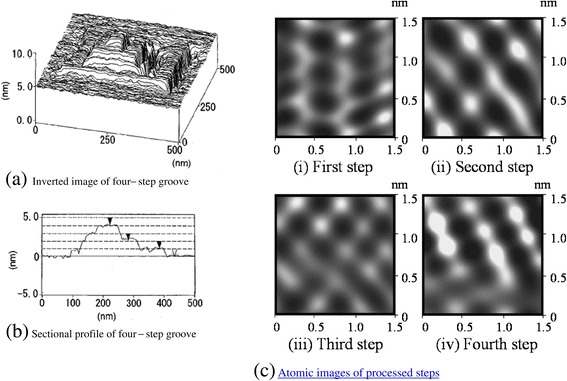
**Processed four-step square groove profile.** Inverted image **(a)** and sectional profile **(b)** of the four-step groove; atomic images of processed steps **(c)**.

Next, processing of the other three steps was performed. The inverted image of the atomic-scale four-step square groove is shown in Figure 13a. In these additional processing steps, the load was 130 nN. Each processing contained five scans, and the scan areas were 200 × 200 nm^2^, 150 × 150 nm^2^, and 50 × 50 nm^2^. Figure 13a is inverted to clearly show the bottoms of the groove; a four-step square groove was obtained. From the cross-sectional profile of this groove (Figure 13b), the depths of the 350 × 350 nm^2^, 200 × 200 nm^2^, 150 × 150 nm^2^, and 50 × 50 nm^2^ scan areas were 1 nm, 2 nm, 3 nm, and 4 nm, respectively. These results show that the processing depth at each step was 1 nm, corresponding to the distance between the cleavage planes.

The surface of each step was rough because of surface adsorbates; however, the surfaces were mainly composed of a basal plane of SiO_4_. Except in the fourth step of the groove, all atomic force distributions had the same periodicity as that of the hexagons formed by SiO_4_ tetrahedra (Figure 13c). The measured periodicity of the surface profile was nearly 0.5 nm, which agreed well with the expected atomic interval periodicity of 0.52 nm. The periodicity of the fourth step slightly contained half that of other steps. Noise and error including double tip imaging appeared to cause the change in periodicity. These results show that the atomic-scale mechanical processing of a four-step groove in a layered crystalline structure can be achieved using AFM.

The atomic-scale mechanical processing of layered materials such as muscovite was performed using AFM. Processing was started at a critical load above 130 nN, and the processing depth increased discretely with load. With a load slightly larger than the critical load, several repetitions of mechanical tip sliding generated a 1-nm-deep groove. This depth corresponds to the distance from the top surface of SiO_4_ to that of the next SiO_4_ layer beneath it, given the removal of residual potassium on the surface. Therefore, the processing was performed with a depth of 1 nm, which corresponds to the layer unit of mica. Furthermore, a groove with four steps of 1-nm depths was processed by step-by-step mechanical sliding.

### Nanoscale line, space, groove, and bending processing of layered crystalline materials

#### Nanoindentation hardness of layered crystalline materials

**Figure 14:**
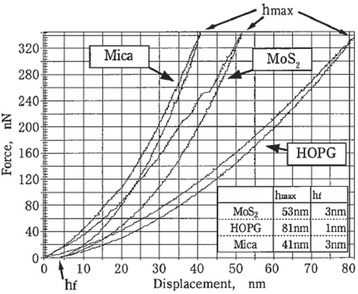
**Nanoindentaion curves of layered crystalline materials (mica, MoS**_2_ **, and HOPG).**

Nanoindentation tests of various multilayered crystalline specimens were conducted using a diamond indenter. Nanoindentation curves (force curves) showing the relationship between the load and the diamond indenter indentation depth are shown in Figure 14. The maximum indentation depth (*h*_max_) is smallest for mica, slightly larger for MoS_2_, and largest for HOPG. The order of the maximum indentation depths of these layered materials corresponds to the order of the cleavage plane periodic length (Figure 4). From the nanoindentation curves, hardness and elastic modulus were evaluated from the tangential lines in the nanoindentation unloading curves. These lines were drawn from the points of maximum depth, and Young’s modulus was calculated from the slopes of the curves. Hardness was calculated on the basis of the intersection depth of those tangential lines and the x-axis [25,28].

**Figure 15:**
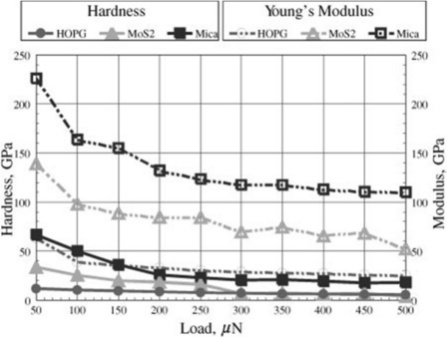
**Hardness and Young’s modulus values evaluated by nanoindentation tests.**

The hardness and Young’s modulus of each layered crystalline material are shown in Figure 15. The hardness of HOPG is low. At loads lower than 250 μN, MoS_2_ is harder than HOPG. On the other hand, HOPG is harder than MoS_2_ at loads higher than 250 μN. The elastic modulus of HOPG is the lowest, whereas that of mica is the highest. The elastic modulus of MoS_2_ is the second highest after that of mica. As the applied load increases, the elastic modulus of MoS_2_ decreases.

**Figure 16:**
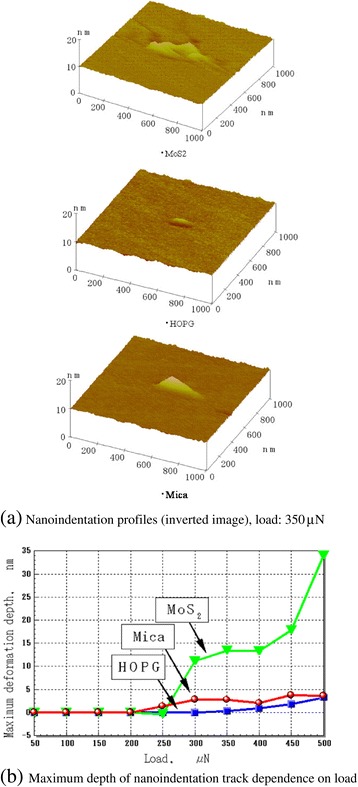
**Evaluation of nanoindentation tracks of layered crystalline materials.** Nanoindentation profiles **(a)** and maximum depth **(b)** of nanoindentation track dependence on load.

The indentation track corresponding to permanent deformation by a diamond tip is shown in Figure 16a. Observation of the indentation depth in layered crystalline materials revealed that the maximum indentation depth (*h*_max_) of HOPG is the largest, followed by MoS_2_ and mica (Figure 14). In contrast, the final deformation depth (*h*_f_; Figure 14) and the actual indentation track depth of HOPG (Figure 16b) were the smallest. The small indentation track size of HOPG indicates that the deformation was mainly elastic. HOPG has a low hardness and a low elastic modulus; however, it is difficult to form permanent indentation tracks. HOPG undergoes negligible plastic deformation but shows some degree of elastic deformation because its basal plane is very strong, and the period of the basal plane is short (Figure 4b).

The maximum track depth of MoS_2_ rapidly increases with load at and above 250 μN (Figure 16b). At loads lower than 250 μN, MoS_2_ has a small indentation depth, and its plastic deformation is not easily observed. Because the basal plane bonds of MoS_2_ are strong compared to those of HOPG, it can be concluded that the fine destruction of the MoS_2_ basal plane does not occur, and a larger damage size is produced in MoS_2_ during the indentation process.

### Processing property dependence on load and sliding cycle

**Figure 17:**
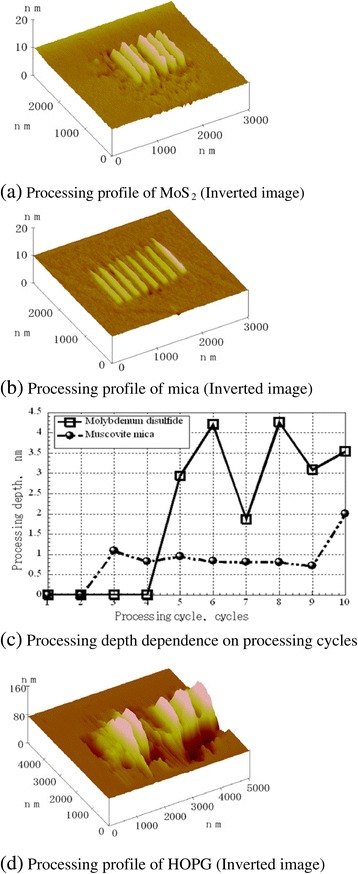
**Dependence of processing depth on number of processing cycles.** Processing profile of MoS_2_ **(a)**, mica **(b)**, and HOPG **(d)**; processing depth dependence on processing cycles **(c)**.

The dependence of the processing results on the number of sliding cycles with multiple repetitions of processing of the same part of various specimens is shown in Figure 17. Inverted images of MoS_2_ and mica processing profiles are shown in Figure 17a,b, respectively. The processing depth dependence on the number of sliding cycles of MoS_2_ and mica is shown in Figure 17c.

In all cases, the processing was performed with a diamond-coated tip at a processing load of 40 μN. The same program was utilized for processing MoS_2_ and mica, but the number and profile of the processed tracks were different. The processed track of mica was observed after three processing cycles (Figure 17b,c), and the processing depth was approximately 0.7 to 1 nm from the third to the ninth processing cycle. This depth corresponds to mica’s cleavage plane interval; thus, processing can be performed at each periodic layer unit of mica.

In contrast, the processed tracks of MoS_2_ were formed after five processing cycles (Figure 17a). Processing depth changed discretely with the number of sliding cycles (Figure 17c); therefore, precise processing at a definite depth unit is difficult. The size of processed debris on the processed surface was larger than that of mica because of the larger destruction size in MoS_2_. The processing depth occurred in multiples of 0.6 nm, which corresponds to the layer-period unit of MoS_2_ (Figure 17c). Thus, processing with a cleavage plane interval was achieved.

After attempting to process HOPG at the same load, no distinct processed tracks could be found on its surface. The load for HOPG processing was then increased to 55 μN, the point at which the processing of HOPG became possible. The processed tracks of HOPG were larger than those of mica and MoS_2_, and the wear tracks overlapped and could not be distinguished (Figure 17d), even at the same line intervals as those of the other two materials. These results indicate that the basal plane strength of HOPG is higher, and that fine local destruction does not easily occur until the deformation range is markedly extended. Thus, nanometer-scale processing is difficult for multilayered crystalline materials with strong basal planes such as HOPG.

### Line processing and lateral force

**Figure 18:**
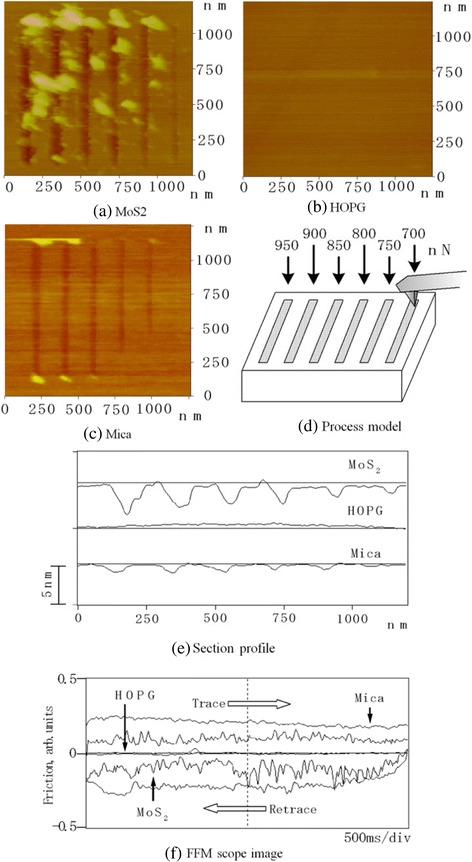
**Processed grooves and friction force.** Processing profile dependence on load **(a, b, c)**; lateral force profiles of various materials at the same load **(d)**; and section profile **(e)** and FFM scope image **(f)**.

The load dependence of the processing profiles and lateral processing force is shown in Figure 8. The processing profile dependence on load is shown in Figure 18a,b,c. In the case of MoS_2_ processing, a large amount of processed debris was observed at the periphery (Figure 18a). Furthermore, the depth suddenly increased with load. A precisely processed profile was observed in mica. Processing debris was scarce in the processed grooves of mica (Figure 8c). In contrast, no processing track was observed for HOPG at the same load (Figure 18b).

Lateral force was measured by FFM during the processing of various specimens. The lateral force profiles of various materials at the same load are shown in Figure 18d. Friction force was evaluated from one cycle of the distance of these trace and retrace curves. Because HOPG was not processed, the lateral force was very small.

The strength of the HOPG basal plane is high; therefore, no fracture occurred due to tip sliding. The processing depth of mica is shallow; however, its lateral force is higher than that of MoS_2_. A comparison of the lateral force wave patterns of MoS_2_ and mica suggests that the greater amount of processing debris and roughness of the processed track observed in MoS_2_ are responsible for the greater lateral force change observed for MoS_2_. Processed surface roughness and change in lateral force seem to be related to the size of destruction by the diamond tip sliding. These results are explained by the larger destruction sizes observed in MoS_2_ processing compared to those in mica due to the higher strength of the MoS_2_ basal plane.

### Processing of nanometer-scale lines, spaces, and lattice grooves

**Figure 19:**
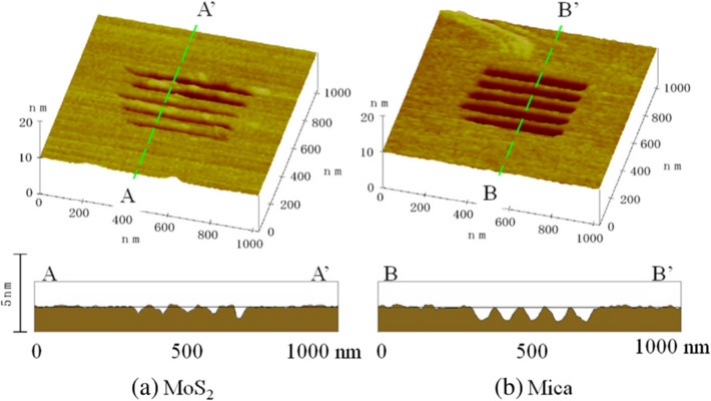
**Lines and spaces processed by DLC-coated tips (load: 1,600 nN; line period: 80 nm).** MoS_2_ **(a)** and mica **(b).**

As an example of nanoprocessing, lines and spaces in MoS_2_ formed by a DLC-film-coated tip under appropriate conditions are shown in Figure 19a. Lines and spaces were processed with a load of 1,600 nN and a line interval of 80 nm by controlling the tip tracks with a nanometer lithographic program. Because an attempt was made to process lines with intervals of less than 50 nm, the processing tracks intersected each other. At this point, the processing depth was about 0.6 nm. This depth corresponds to the 0.616-nm cleavage plane period of MoS_2_. This means that only one layer of the basal plane of MoS_2_ can be processed at this time. Mica can be processed more easily in the 1-nm layer-period unit (Figure 19b). In contrast, even when using a DLC-film-coated tip, the precise nanometer-scale processing of HOPG was impossible because of the high strength of its basal plane.

**Figure 20:**
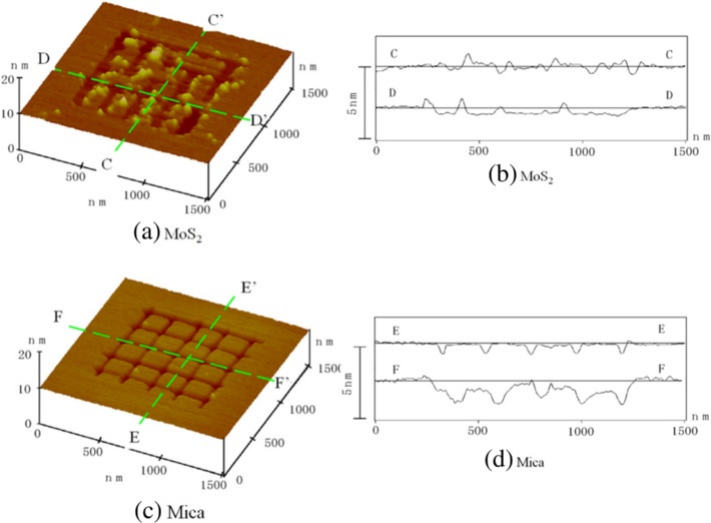
**Cross groove processed by a DLC-coated tip (load: 1,600 nN; line period: 200 nm) (a, b, c, d).**

An example of MoS_2_-processed lattice grooves with 200-nm line intervals is shown in Figure 20. The processing depth of the lines was approximately 0.6 nm. As a result, nearly 100-nm square plates were formed in a 1 × 1 μm^2^ area supported by van der Waals forces. The shapes of the processed grooves in MoS_2_ were not as distinct as those in mica, and visible processing debris was not removed because of the tip scanning performed to measure the shapes. The continuous processing of MoS_2_ in stress-concentrated areas is problematic because its basal plane strength is higher than that of mica. Therefore, the accurate nanometer-scale cutting processing of MoS_2_ is difficult.

### Bending processing for graphite basal planes (graphene)

The nanoscale processing of HOPG is quite difficult because its basal plane is an extremely thin plate supported by van der Waals forces, and the bond strength of the graphite basal plane is high. Therefore, the bending of the basal planes supported by van der Waals forces can be regarded as the bending of nanometer-scale graphene plates; whether the nanometer bending processing of this strong basal plane can be realized is a very interesting problem.

**Figure 21:**
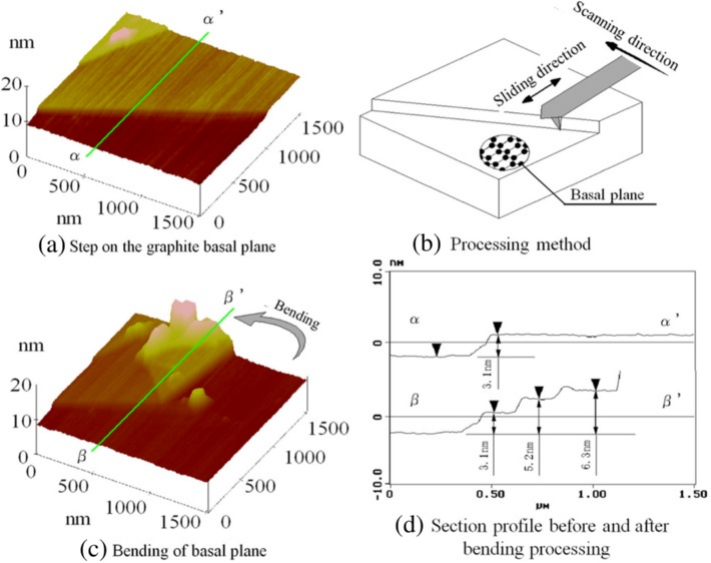
**Bending processing of graphite basal planes by diamond tip sliding.** Three-dimensional general surface image of HOPG **(a)**. Processing method **(b)** and bending of the basal plane **(c)**. Cross-sectional profile before and after the bending process **(d)**.

A three-dimensional general surface image of HOPG is shown in Figure 21a. Steps were observed on the HOPG cleavage plane surfaces. When load was applied to a step on the surface with simultaneous scanning by a diamond tip (Figure 21b), the step was easily deformed (Figure 21c). The HOPG surface, however, was not completely removed. Even with tip scanning, the deformed part of HOPG remained on the surface. It is evident that there was some folding of the HOPG basal plane. From the cross-sectional profile (Figure 21d), the original step height (*α* - *α*′) was 3.1 nm, equivalent to approximately nine layers of the graphite basal plane. The 6.3-nm-thick folding layer (*β* - *β*′) was equivalent to approximately 18 graphene layers. Thus, HOPG basal planes are generally considered to be folding-bending objects that undergo little destruction and removal after a folding process. Even though the tip used in observing the shapes scans a folding part, the folding part is unchanged and maintains the processed folding profiles.

After attempting the same nanobending processing for mica and MoS_2_, removal deformation was achieved. The phenomenon of folding deformation was not observed in these materials. Moreover, the destruction sizes of mica and MoS_2_ were small because of the lower strengths of their basal planes. The above results demonstrate that the nanobending processing of several plates supported by van der Waals forces can be realized because the graphene strength of graphite is very high.

## Conclusions

We described atomic-scale nanoprocessing technologies for layered crystalline materials, such as the characterization of atomic-scale wear properties and the 1-nm-deep mechanical processing of muscovite by AFM. Mechanical processing properties of the layered crystalline materials were also determined by AFM. The described process details clearly showed that materials with layered crystalline structures such as MoS_2_ and mica have weakly interacting basal planes; therefore, the surfaces of these materials are easily cleaved at the basal plane with atomic-scale precision by the mechanical action of a tip. We hope that these descriptions will contribute to the further development of this research area.
